# Hepatic decompensation during paritaprevir/ritonavir/ombitasvir/dasabuvir treatment for genotype 1b chronic hepatitis C patients with advanced fibrosis and compensated cirrhosis

**DOI:** 10.1371/journal.pone.0202777

**Published:** 2018-08-23

**Authors:** Yi-Chung Hsieh, Wen-Juei Jeng, Chien-Hao Huang, Wei Teng, Wei-Ting Chen, Yi-Cheng Chen, Shi-Ming Lin, Dar-In Tai, Chun-Yen Lin, I-Shyan Sheen

**Affiliations:** 1 Division of Hepatology, Department of HepatoGastroenterology, Chang-Gung Memorial Hospital, Linkou Medical Center, Taoyuan, Taiwan; 2 School of Medicine, College of Medicine, Chang-Gung University, Taoyuan, Taiwan; 3 School of Traditional Chinese Medicine, College of Medicine, Change-Gung University, Taoyuan, Taiwan; National Taiwan University Hospital, TAIWAN

## Abstract

**Background and aim:**

Hepatic decompensation is a severe on-treatment adverse event for chronic hepatitis C treated with paritaprevir/ritonavir/ombitasvir and dasabuvir (PrOD). Till now, few papers regarding on-treatment hepatic decompensation have been reported. The study aims to analyze the general feature and predictive factors of on-treatment hepatic decompensation in hepatitis C virus (HCV) genotype 1b-infected patients with advanced fibrosis and compensated cirrhosis who receive treatment with PrOD.

**Methods:**

A real-word cohort enrolled 189 HCV genotype 1b patients with advanced fibrosis and compensated cirrhosis treated with 12-week PrOD. Clinical and laboratory data were analyzed between patients with and without on-treatment hepatic decompensation.

**Results:**

The sustained virologic response rate at 12 weeks after treatment was 97.3% in HCV subtype 1b patients with advanced fibrosis and cirrhosis. On-treatment hyperbilirubinemia (total bilirubin >2 mg/dL) occurred in 27 (14.3%) patients, and the incidence of the increase of total and direct form bilirubin was significantly different during treatment between patients with Child-Turcotte-Pugh score 5 and score 6. Five (18.5%) hyperbilirubinemia patients progressed to hepatic decompensation. Older age (adjusted OR: 1.2, 95% CI: 1.0–1.4) and albumin ≤3.6 g/dL (adjusted OR: 10.4, 95% CI: 1.3–81.2) may be two predictors for on-treatment hepatic decompensation by multivariate analysis.

**Conclusions:**

PrOD is an effective direct-acting antiviral agent for antiviral therapy in HCV genotype 1b patients with advanced fibrosis and cirrhosis. Hyperbilirubinemia is possibly the early warning feature of on-treatment hepatic decompensation. This serious adverse event of on-treatment hepatic decompensation is not common. Older age and low baseline albumin level may be predictive factors.

## Introduction

Chronic hepatitis C virus (HCV) infection is a major health issue worldwide, and accounts for serous complications including liver cirrhosis, liver failure, and hepatocellular carcinoma (HCC), leading to around 700,000 deaths annually [1–3]. The eradication of HCV by antiviral therapy has been proved to significantly reduce all-cause and liver-related mortality, and this is especially important in patients with advanced fibrosis and cirrhosis to halt disease progression [4–8]. The stand of care for chronic hepatitis C (CHC) shifted gradually from the dual therapy with pegylated interferon (PEG-IFN) and ribavirin (RBV) to interferon (IFN)-free direct-acting antiviral agents (DAAs) since 2011, and the overall sustained virologic response (SVR) rates increased from 55% to nearly 100% [9–11].

Paritaprevir/ritonavir/ombitasvir plus dasabuvir (PrOD) is one of very effective DAAs for HCV genotype 1, which inhibits the function of nonstructural (NS) 3/4A protease, NS5A protein, and NS5B ribonucleic acid (RNA)-dependent polymerase, and ever occupied considerable market share [2, 7, 12, 13]. However, warning of severe liver injury, hepatic decompensation and even death during the treatment were reported and informed by US Food and Drug Administration (FDA) in 2015 [14–18]. Further, in 2017, the American Association for the Study of Liver Diseases and the infectious diseases society of America (AASLD-IDSA) HCV guidance gave a definite recommendation to avoid PrOD-induced hepatic adverse events [7].

Hepatic decompensation is a severe and most dangerous complication during the course of DAA therapy, and also a prelude to liver failure and mortality. It is particularly catastrophic when hepatic decompensation happened in patients with advanced fibrosis and cirrhosis. An unmet need is to establish objective criteria and the general feature of hepatic decompensation so that either supportive care or early discontinuation of DAA during antiviral treatment can be determined [19]. Although AASLD-IDSA HCV guidance suggests to closely monitor lab or clinical symptoms in patients with “advanced liver disease” during the PrOD-based treatment, messages about on-treatment hepatic decompensation with PrOD are few, and there is even no real-world report in the group of HCV subtype 1b and advanced fibrosis and compensated cirrhosis in Asia.

We therefore conducted this prospective study analyzing the real-world cohort to elucidate the predictors of on-treatment hepatic decompensation in HCV genotype 1b-infected patients treated by the PrOD regimen in the setting of advanced fibrosis and liver cirrhosis.

## Materials and methods

### Patients

**Fig 1:**
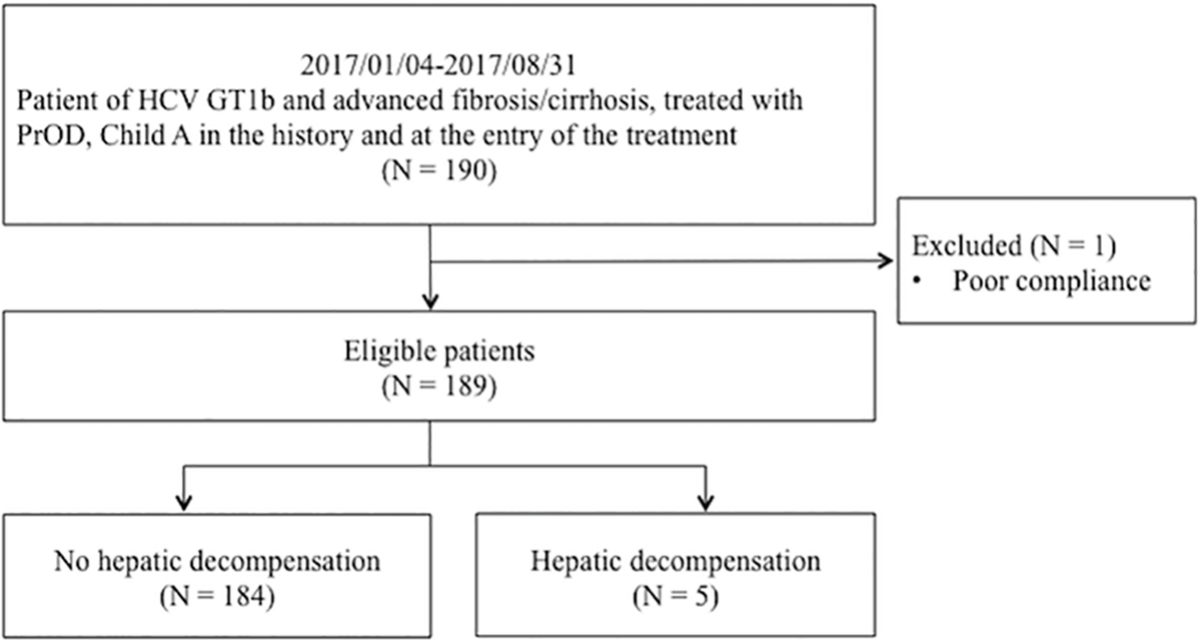
The selection of patients included in the study. HCV GT1b = hepatitis C virus genotype 1b, PrOD = paritaprevir/ritonavir/ombitasvir plus dasabuvir.

This prospective real-world cohort recruited chronic HCV infected patients who received antiviral treatments reimbursed by the Taiwan National Health Insurance Administration in Chang Gung Memorial hospital, Linkou Medical center between January 4, 2017 and August 31, 2017. The eligibility criteria included HCV genotype 1b, advanced fibrosis or compensated liver cirrhosis [Child-Turcotte-Pugh (CTP) class A] at the entry of the treatment, and treatment with PrOD (Fig 1). The study protocol adhered to the ethical guideline of the 1975 Declaration of Helsinki and was approved by the ethical committees of Chang Gung Memorial Hospital.

The IFN/RBV-free antiviral treatment consisted of a 12-week oral coformulated PrOD (paritaprevir/ritonavir/ombitasvir 150 mg/100 mg/25 mg once daily, and dasabuvir 250 mg twice daily). HCV genotype and subtype were identified prior to treatment. Hemogram, prothrombin time (PT) and international normalized ratio (INR), and a liver function panel were assayed every 1–2 weeks at the first 4 weeks of therapy, and thereafter every 2–4 weeks till the end of treatment (EOT). HCV RNA was measured at baseline, week 4 of therapy, EOT, and 12 weeks after EOT.

The stage of hepatic fibrosis was determined by liver biopsy, transient elastography (FibroScan), acoustic radiation force impulse elastography (ARFI), Fibrosis-4 (FIB-4) score, or abdominal ultrasonography. In patients with hepatic fibrosis stage 3 (F3), 24 (21.4%) were staged by liver biopsy, 65 (58.0%) by FibroScan, 2 (2.8%) by ARFI, and 21 (18.8%) by FIB-4 score. In patients with hepatic fibrosis stage 4 (F4), 10 (13.0%) were staged by liver biopsy, 35 (45.5%) by FibroScan, 2 (2.6%) by ARFI, 27 (35.1%) by FIB-4 score, and 3 (3.9%) by abdominal ultrasonography. Liver biopsies were evaluated by an experienced hepatopathologist using the METAVIR scoring system in which F3 and F4 were considered as advanced fibrosis and liver cirrhosis, respectively. The diagnosis of liver cirrhosis by abdominal ultrasonography was based on heterogenous/coarse liver parenchyma with uneven surface plus gastroesophageal varices or/and splenomegaly.

In this study, we modified the US FDA warning and the content of the AASLD-IDSA guideline, and defined “hepatic decompensation” as (a) signs of worsening liver disease [ascites, variceal hemorrhage, encephalopathy, PT prolong or INR increase, or/and deteriorated CTP class (from class A to B or C)], or/and (b) significantly increased bilirubin (total form >3 mg/dL and direct form >30%) [7, 18]. EOT virologic response (EOTVR) was defined as undetectable HCV RNA at EOT. SVR12 was defined as HCV RNA <15 IU/mL 12 weeks after EOT.

The HCV antibody was tested by the Architect anti-HCV (Abbott Diagnostics, Irving, TX, USA). The HCV RNA level was measured by a commercial quantitative polymerase chain reaction (PCR) assay: VERSANT HCV RNA 3.0 assay (HCV 3.0 bDNA assay, Bayer Diagnostics, Berkeley, CA, USA, lower limit of detection: 5.2 x 10^2^ IU/mL) or COBAS TaqMan HCV Test (TaqMan HCV; Roche Molecular Systems Inc., Branchburg, NJ, USA, lower limit of detection: 15 IU/mL). If VERSANT HCV RNA 3.0 assay showed undetectable HCV RNA, HCV RNA would be tested again by COBAS AMPLICOR HCV test, v2.0 (CA V2.0, Roche Diagnostic Systems, Pleasanton, CA, USA, lower limit of detection: 50 IU/ mL). The HCV genotype was determined using a genotype-specific probe-based assay in the 5’ untranslated region (LiPA; Innogenetics, Ghent, Belgium).

### Statistical analysis

The difference of clinical characteristics between patients with and without liver decompensation was compared using the independent student t-test or Mann-Whitney U test for continuous variables according to if normal distribution, and using Chi-square test or Fisher’s exact test for categorical variables. Univariate and multivariate binary logistic regression analyses were conducted for the predictors of hepatic decompensation. P values <0.05 by the two-tailed test were considered statistically significant. A receiver operating characteristic (ROC) curve was applied to find out the maximal area under the ROC (AUROC), and the best cut-off point for liver decompensation was determined by the maximum value of Youden’s index. All statistical analyses were done with the statistical software, IBM SPSS Statistics Version 20.

## Results

### Patients demographics

Total 189 HCV genotype 1b-infected patients with advanced fibrosis and liver cirrhosis treated by PrOD were enrolled in this study (Fig 1). The mean age was 65.2±9.1 years old, and 78 (41.3%) were male. The majority of patients were treatment-experienced (N = 151, 79.9%). CTP scores at entry were mainly score 5 (N = 181, 95.8%) while the rest were score 6. Seventy-seven (40.7%) patients were diagnosed with the METAVIR fibrosis stage of F3 and the others were F4. Forty-eight (25.4%) had HCC, and 36 (75.0%) were cure and 9 (18.8%) had active HCC at the time of PrOD treatment. Baseline biochemistry showed mean albumin level was 4.2±0.4 g/dL, mean alanine aminotransferase (ALT) level was 96±59 U/L, mean total/direct/indirect bilirubin levels were 0.84±0.33/0.33±0.18/0.53±0.28 mg/dL, mean international normalized ratio (INR) was 1.10±0.08, and mean FIB-4 score was 5.62±3.98. Mean HCV RNA level was 6.22±0.59 log_10_IU/mL. EOTVR rate was 97.4%, and SVR12 rate was 97.3% (Table 1).

**Table 1:** Clinical characteristics of patients treated with PrOD.

Characteristic	Total	Hepatic decompensation		P value
	(n = 189)	No (n = 184)	Yes (n = 5)	
Age, years	65.2±9.1	64.9±8.9	78.3±6.9	0.001
Gender (male), %	78 (41.3)	76 (41.3)	2 (40.0)	1.0
Treatment-experienced, %	151 (79.9)	148 (80.4)	3 (60.0)	0.264
CTP at entry, %				0.196
5	181 (95.8)	177 (96.2)	4 (80.0)	
6	8 (4.2)	7 (3.8)	1 (20.0)	
METAVIR score				1.000
F3	77 (40.7)	75 (40.8)	2 (40.0)	
F4	112 (59.3)	109 (59.2)	3 (60.0)	
HCC status at entry, %	48 (25.4)	45 (24.5)	3 (60.0)	0.105
Cure	36 (19.0)	33 (17.9)	3 (60.0)	
Active	9 (4.8)	9 (4.9)	0 (0)	
Baseline data				
Biochemistry				
Albumin (g/dL)	4.2±0.4	4.2±0.4	3.6±0.1	< 0.001
>3.6	172 (91.0)	170 (92.4)	2 (30.0)	0.005
≤3.6	17 (9.0)	14 (7.6)	6 (60.0)	
AST (U/L)	88±49	89±53	70±27	0.448
ALT (U/L)	96±59	98±65	54±15	0.134
Bilirubin (mg/dL)				
Total form	0.84±0.33	0.84±0.33	0.86±0.11	0.939
Direct form	0.33±0.18	0.33±0.18	0.33±0.05	0.981
Indirect form	0.53±0.28	0.53±0.29	0.53±0.13	0.991
Platelet (1000/μL)	129±53	129±53	141±74	0.618
INR	1.10±0.08	1.10±0.08	1.12±0.04	0.622
FIB-4	5.62±3.98	5.36±3.75	6.22±3.22	0.581
HCV RNA (log_10_ IU/mL)	6.22±0.59	6.23±0.59	5.84±0.38	0.138
EOTVR, %	184 (97.4)	184 (100)	NA	
SVR12, %	109/112 (97.3)	107/109 (98.2)	2/3 (66.7)	0.079

PrOD = paritaprevir/ritonavir/ombitasvir plus dasabuvir; CTP = Child-Turcotte-Pugh score; AST = aspartate aminotransferase; ALT = alanine transaminase; INR = international normalized ratio; FIB-4 = Fibrosis-4 score; HCC = hepatocellular carcinoma; HCV RNA = hepatitis C virus ribonucleic acid; EOTVR = end-of-treatment virologic response; SVR12 = sustained virologic response at 12 weeks after treatment; NA = not available.

### On-treatment hyperbilirubinemia

**Fig 2:**
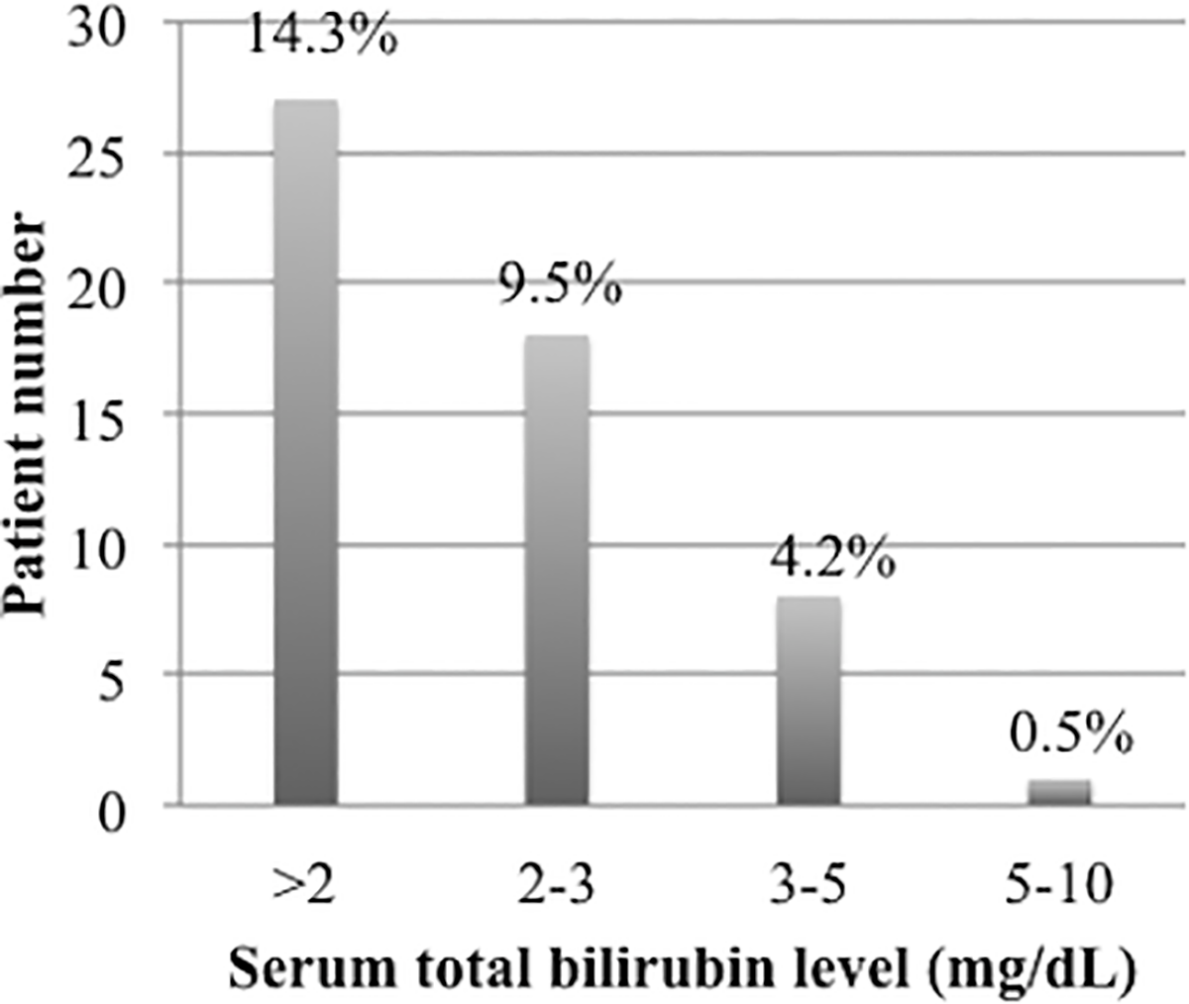
Hyperbilirubinemia (total bilirubin >2 mg/dL) during the treatment.

**Fig 3:**
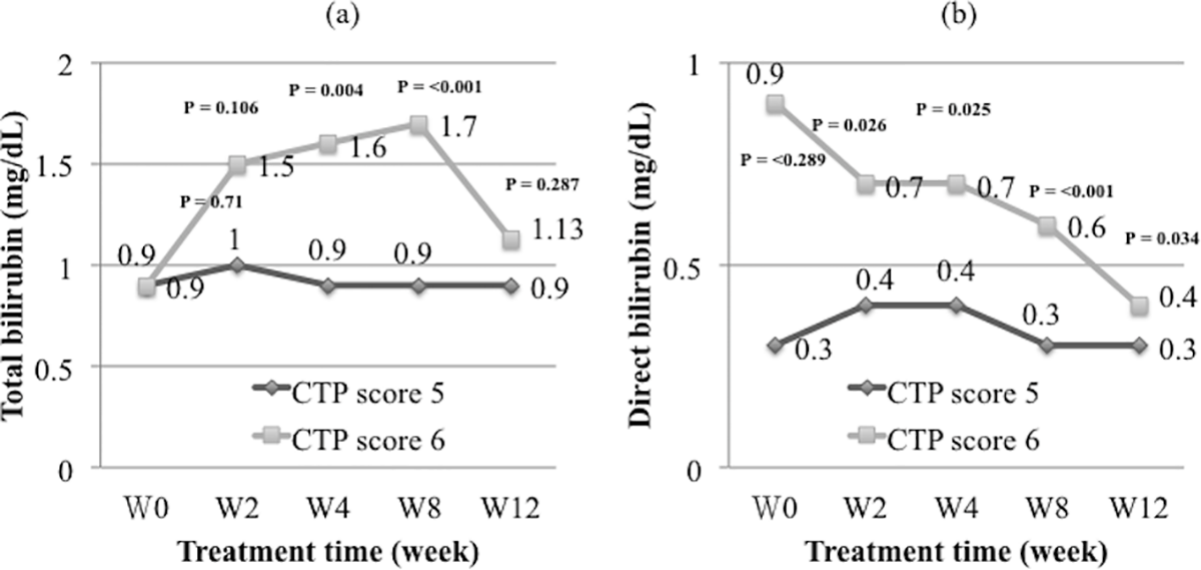
(a) Total bilirubin elevation during the treatment. (b) Direct form bilirubin elevation during the treatment.

During the treatment with PrOD, the level of total bilirubin >2 mg/dL happened in 27 (14.3%) patients, in whom 9 patients (33.3%) had serum total bilirubin level exceeding 3 mg/dL (Fig 2). Among 27 hyperbilirubinemia (total bilirubin >2mg/dL) patients, only 19 (70.4%) had complete and corresponded direct form bilirubin, and they all had conjugated hyperbilirubinemia (direct form bilirubin >0.4 mg/dL based on the reference of our hospital). The level of serum total bilirubin was significantly higher in the group of CTP score 6, in contrast to CTP score 5, at treatment week 4 and week 8; the level of direct bilirubin was significantly higher in the group of CTP score 6 at treatment week 2, week 4, week 8, and week 12. Five out of 27 (18.5%) hyperbilirubinemia (total bilirubin >2 mg/dL) patients progressed to hepatic decompensation, and the remainder recovered to normal at EOT (Fig 3).

### On-treatment hepatic decompensation

Five (2.65%) patients suffered from hepatic decompensation during the treatment of PrOD and finally withdrew antiviral therapy. All 5 people were older than 72 years old. Among these 5 patients, 4 (80%) were female, 3 (60%) were treatment-experienced, 4 (80%) had cirrhotic liver, 3 (60%) had HCC [all 3 (100%) had previously cured HCC before the PrOD treatment], and only 1 (20%) had a viral load more than 6 log_10_ IU/mL. All 5 (100%) patients underwent elevated bilirubin greater than 2 mg/dL without prominent increases in aminotransferases. Two patients had SVR12 but one hadn’t, and the residual two can’t have sufficient follow-up time to confirm SVR12. Four (80%) patients withdrew antiviral treatment due to hepatic decompensation with signs of worsening liver disease and significantly increased bilirubin. Only one (20%) ceased the treatment by week 8 of therapy due to the presence of hepatic encephalopathy and deteriorated CTP class though the bilirubin level was 2.3 mg/dL (Table 2).

**Table 2:** Characteristics of 5 patients with on-treatment hepatic decompensation.

	Case 1	Case 2	Case 3	Case 4	Case 5
Age, years	76.4	80.1	73.3	89.4	72.2
Gender	M	F	F	F	F
Treatment-experienced	Yes	Yes	Yes	No	No
Liver cirrhosis	Yes	Yes	Yes	No	Yes
HCC	No	Yes	Yes	No	Yes
Cure	-	Yes	Yes	-	Yes
Baseline data					
HCV RNA (log_10_ IU/mL)	5.63	5.90	5.74	6.45	5.47
Albumin (g/L)	3.85	3.54	3.61	3.56	3.49
AST (U/L)	55	104	89	36	68
ALT (U/L)	57	59	73	33	47
Bilirubin (mg/dL)					
Total form	0.9	0.9	1.0	0.7	0.8
Direct form	-	0.4	0.3	0.3	0.3
Indirect form	-	0.5	0.7	0.4	0.5
INR	1.1	1.1	1.1	1.2	1.1
Platelet (1000/μL)	255	158	74	143	76
Data to stop PrOD during treatment					
Maximum total/direct bilirubin (mg/dL)	3.0/-	4.4/2.4	2.3/-	4.7/2.3	5.1/2.1
AST (U/L)	52	140	70	28	98
ALT (U/L)	54	54	95	21	79
Length of treatment	2W	9W	8W	<1W	4W
SVR	NA	NA	Yes	No	Yes
Features of hepatic decompensation to withdraw PrOD treatment					
	A + B	A + B	A	A + B	A + B

M = male; F = female; HCC = hepatocellular carcinoma; HCV RNA = hepatitis C virus ribonucleic acid; AST = aspartate aminotransferase; ALT = alanine transaminase; INR = international normalized ratio; PrOD = paritaprevir/ritonavir/ombitasvir plus dasabuvir; W = week; SVR = sustained virologic response; NA = not available; A = signs of worsening liver disease; B = significantly increased bilirubin.

#### Predictive factors for on-treatment hepatic decompensation

**Fig 4:**
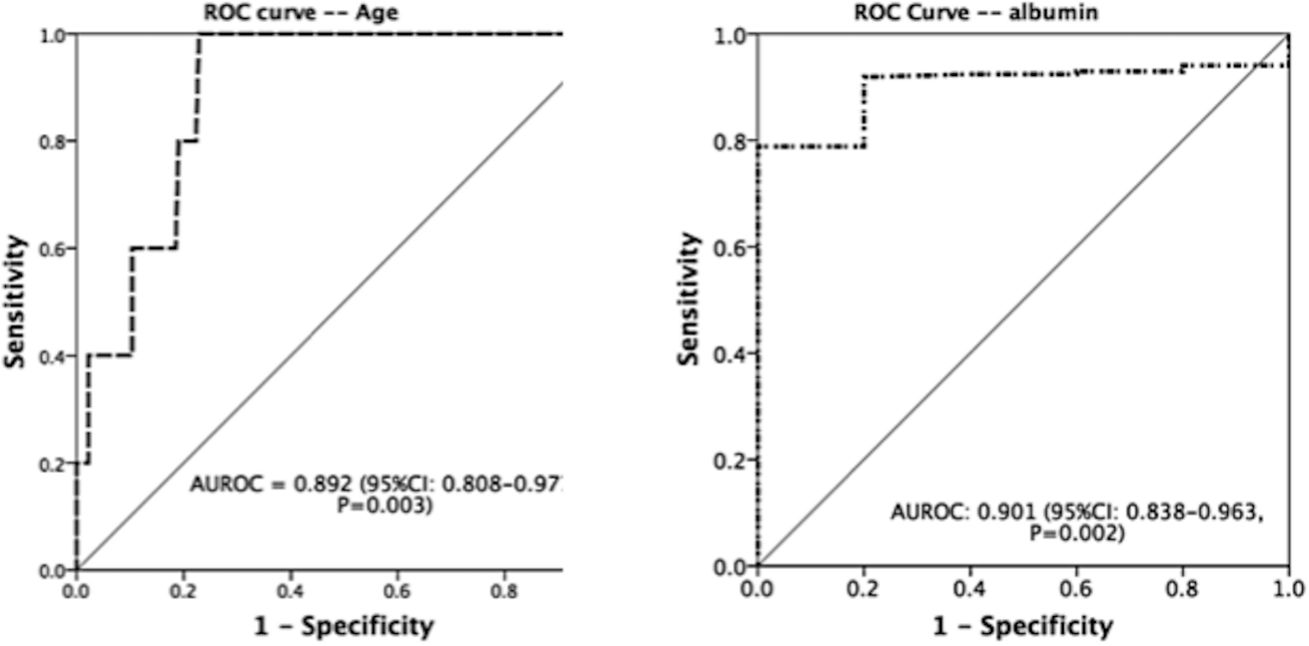
ROC curve for age and albumin in predicting hepatic decompensation.

Based on univariate analyses, the significant differences between two groups of patients with and without hepatic decompensation were age (OR 1.22, 95% CI: 1.07–1.40, p = 0.003) and albumin ≤3.6 g/dL (OR 18.21, 95% CI: 2.81–118.22, p = 0.005) (Table 1). The ROC curve was used to get the best cut-off values of age and albumin for hepatic decompensation (Fig 4). The age of 71 years old was associated with maximal AUROC of 0.892 (p = 0.003, 95% CI: 0.81–0.98, sensitivity 100%, specificity 75%), and the level of albumin 3.6 g/dL met maximal AUROC of 0.901 (p = 0.002, 95% CI: 0.84–0.96, sensitivity 80%, specificity 92%).

By multivariate analysis, age (p = 0.015, OR: 1.19, 95% CI: 1.04–1.36) and albumin ≤3.6 g/dL (p = 0.026, OR: 10.37, 95% CI: 1.33–81.20) were two independent predictive factors for on-treatment hepatic decompensation (Table 3).

**Table 3:** Logistic regression analysis of predictors for on-treatment hepatic decompensation.

Variable	Adjusted OR	95% CI of OR	P value
Age, years	1.19	1.04–1.36	0.015
Albumin ≤3.6 (g/dL)	10.372	1.33–81.20	0.026

OR = odds ratio; CI = confidence interval.

## Discussion

The treatment-emergent hepatic adverse events are the major drawback of DAA treatment for CHC though high successful rates. Among them, hepatic decompensation had come into public notice by reason of its possibility for consequent liver failure that may lead to liver transplantation and mortality [19]. Thus, AASLD-IDSA guidance proposed patients with current or previous decompensated liver disease (CTP score ≥7) should not received NS3 protease inhibitors (i.e., paritaprevir)–containing regimen because of on-treatment liver injury [7]. In this real-world study, we reported SVR12 rate in CHC genotype 1b patients with advanced fibrosis and compensated cirrhosis was 97.3%, similar to other Western and Asian studies [20–28]. A small proportion (14.3%) of patients underwent on-treatment hyperbilirubinemia (total bilirubin >2 mg/dL), and five of them progressed to hepatic decompensation. The old age and low albumin were the risk factors for the development of hepatic decompensation. To the best of our knowledge, this is the 1^st^ Asian real-world experience study addressed hepatic decompensation under treatment with PrOD focused on HCV genotype 1b-infected patients with advanced fibrosis and compensated cirrhosis.

Previous studies of hepatic decompensation during PrOD treatment were mainly obtained from clinical trials, and they and our study were summarized in S1 Table. In people with HCV genotype 1 and 4, a pooled analysis of phase II/III clinical trials on CTP A cirrhosis exhibited 1.2% patients had adverse effects consistent with hepatic decompensation, and another recently published meta-analysis showed the rate of hepatic decompensation was <1% across more than 3000 patients, 70% of which had cirrhosis [22, 25]. In a real-world experience for genotype 1 and 4 in Poland, Flisiak et al. had reported an incidence of 3.3% of the event of hepatic decompensation in 209 patients of which 56.9% had liver cirrhosis [20]. With regard to genotype 1-infected patients, an observation from Australia had displayed the occurrence rate of hepatic decompensation was 2.7% in 451 patients with 75.4% being cirrhotic [28]. For patients with subtype 1b infection, a study from Romania reported an incidence of liver decompensation of 1.9% out of 2070 advanced fibrotic patients [23]. However, there were only two real-life reports in Asia mentioning hepatic adverse events. One study from Hong Kong described no on-treatment hepatic decompensation happened among 41 patients (85% genotype 1 and 61% compensated liver cirrhosis), but the smaller sample size was noted [29]. Another retrospective analysis of PrOD-based treatment in HCV genotype 1b was from Taiwan and had shown only one out of 103 (1%) patients developed liver decompensation. However, in this study, only 49.3% of patients were with advanced fibrosis [27].

It was reported that low platelet count, increased total bilirubin, prolonged INR and low albumin were the risk factors of on-treatment liver decompensation in subtype 1b [23]. Another study composed of genotype 1 patients had shown the risk factors were low platelet count and low albumin [28]. The other analysis from HCV genotype 1 and 4 patients demonstrated that lower baseline albumin, prior history of non-selective beta-blocker use and lower baseline HCV RNA were factors associated with liver decompensation [22]. In our study, the rate of incidence of hyperbilirubinemia (total bilirubin >2 mg/dL) and hepatic decompensation during treatment were 14.3% and 2.65%, respectively. The old age and low albumin were the independent risk factors for the development of hepatic decompensation, compatible with other reports. Moreover, all patients with on-treatment hepatic decompensation were older than 70 years old, and this reflected that treated patients were older in Asia than in Western countries.

Isolated hyperbilirubinemia during PrOD therapy without accompanied elevation of serum aminotransferase is a unique phenomenon prior to the occurrence of hepatic decompensation [19]. This is quite different from the other protease inhibitor of asunaprevir in which the serum aminotransferase elevation was associated with the increased total bilirubin level [24]. For this reason, we had provided the definite criteria for hepatic decompensation in this paper, and intensified significantly hyperbilirubinemia as one of two cardinal components. After further evaluating the elevation of total bilirubin in our study, we found this usually happened in the first 4 weeks and remarkably more frequently in patients with CTP score 6 than score 5. This observation implied this special on-treatment isolated hyperbilirubinemia is possibly related to the underlying liver functional reserve. Among advanced fibrotic and cirrhotic individuals with on-treatment hyperbilirubinemia (total bilirubin >2 mg/dL), 18.5% advanced to hepatic decompensation. Conjugated hyperbilirubinemia is probably the early feature of decompensation that heightened vigilance.

There are two limitations in our study. First, we might overestimate hepatic fibrosis stages. The severity of liver fibrosis was assessed in different ways, that is, liver biopsy or non-invasive alternatives. This approach might result in inconsistency in the evaluation of fibrosis stage. Second, this real-world cohort was relatively few. Only five cases encountered liver decompensation, and this could result in relatively low power for statistical analysis.

In summary, our real-world experience revealed that PrOD treatment is a quite effective treatment for HCV genotype 1b patients with advanced fibrosis and compensated cirrhosis. Hyperbilirubinemia is possibly the early feature of on-treatment hepatic decompensation. The serious adverse event such as hepatic decompensation is not common but should be cautious when the PrOD regimen is applied for those patients with older age and low serum albumin. Closely monitoring serum total bilirubin level during PrOD therapy may be helpful to early detection of liver adverse event and avoiding hepatic decompensation.

## Supporting information

S1 Table Summary of studies about on-treatment hepatic decompensation with PrOD. (DOCX)
