# Supplementary material for: Hepatic decompensation during paritaprevir/ritonavir/ombitasvir/dasabuvir treatment for genotype 1b chronic hepatitis C patients with advanced fibrosis and compensated cirrhosis
Source: PLoS One. 2018 Aug 23;13(8):e0202777. doi: 10.1371/journal.pone.0202777 (PMC6107263; doi:10.1371/journal.pone.0202777)
Supplement: S1 Table — (DOCX) [file pone.0202777.s001.docx]

**Supporting Information Table 1. Summary of studies about on-treatment hepatic decompensation with PrOD**

**Supporting Information Table 1.** Summary of studies about on-treatment hepatic decompensation with PrOD

| Source | Year | DAA | Genotype | Patient number | Advanced fibrosis | Liver cirrhosis | SVR12 | Decompensation |
| --- | --- | --- | --- | --- | --- | --- | --- | --- |
| Poordad^22^ | 2017 | 2D/3D +/- RBV | 1, 4 | 1066 | 0% | 100% | - | 1.2% |
| Wedemeyer^25^ | 2017 | 2D/3D +/- RBV | 1, 4 | 3440 | - | 70% | 97% | <1% |
| Flisiak^20^ | 2016 | PrOD +/- RBV | 1, 4 | 209 | - | 56.9% | 99.0% | 3.3% |
| Lubel^28^ | 2017 | PrOD +/-RBV | 1 | 451 | - | 75.4% | 95.1% | 2.7% |
| Preda^23^ | 2017 | PrOD + RBV | 1b | 2070 | 0% | 100% | 96.6% | 1.9% |
| Chan HL^29^ | 2017 | PrOD +/- RBV | 1 | 41 | - | 61% | 95% | 0% |
| Liu CH^27^ | 2018 | PrOD +/- RBV | 1b | 103 | 18.4% | 31.1% | 98.1% | 1% |
| Hsieh YC | 2018 | PrOD | 1b | 189 | 40.7% | 59.3% | 97.3% | 2.65% |

DAA: direct-acting antiviral agents; SVR12: undetectable HCV RNA 12 weeks after completion of treatment; 2D: paritaprevir/ritonavir/ombitasvir; 3D: ombitasvir/paritaprevir/ritonavir + dasabuvir; RBV: ribavirin; PrOD: ombitasvir/paritaprevir/ritonavir + dasabuvir
